# The Expression and Functions of Toll-Like Receptors in Atherosclerosis

**DOI:** 10.1155/2010/393946

**Published:** 2010-06-24

**Authors:** Jennifer E. Cole, Ektoras Georgiou, Claudia Monaco

**Affiliations:** Kennedy Institute of Rheumatology, Faculty of Medicine, Imperial College, 65 Aspenlea Road, London W6 8LH, UK

## Abstract

Inflammation drives atherosclerosis. Both immune and resident vascular cell types are involved in the development of atherosclerotic lesions. The phenotype and function of these cells are key in determining the development of lesions. Toll-like receptors are the most characterised innate immune receptors and are responsible for the recognition of exogenous conserved motifs on pathogens, and, potentially, some endogenous molecules. Both endogenous and exogenous TLR agonists may be present in atherosclerotic plaques. Engagement of toll-like receptors on immune and resident vascular cells can affect atherogenesis as signalling downstream of these receptors can elicit proinflammatory cytokine release, lipid uptake, and foam cell formation and activate cells of the adaptive immune system. In this paper, we will describe the expression of TLRs on immune and resident vascular cells, highlight the TLR ligands that may act through TLRs on these cells, and discuss the consequences of TLR activation in atherosclerosis.

## 1. Introduction

Atherosclerosis is the principal cause of coronary artery and cerebrovascular disease, which together comprise the leading cause of death, accounting for a fifth of all deaths worldwide [1]. Over the past decade, a major change has occurred in the understanding of the mechanisms responsible for the development and progression of atherosclerosis, leading to an increasing recognition of atherosclerosis as an “inflammatory disease” [2]. Similarities in cellular and molecular mediators of disease can be found between atherosclerosis and other classical chronic inflammatory diseases, such as rheumatoid arthritis (RA) [3]. Similar to other inflamed tissues such as rheumatoid synovium, the atherosclerotic plaque is characterised by the migration into tissue of blood-borne inflammatory cells, followed by interactions with vascular endothelial cells and connective-tissue cells, leading to a chronic inflammatory response. In support of a strong link between inflammation and cardiovascular disease, RA, is associated with an increased risk of cardiovascular events, which account for 35% to 50% of excess premature mortality in RA patients [4]. 

Endothelial dysfunction/activation, is the earliest step in the pathogenesis of atherosclerosis [2]. Endothelial dysfunction can be induced by numerous factors including cytokines, free radicals, lipids, and bacterial or viral infection. In addition, endothelial cells may be primed for activation by haemodynamic forces. Activated endothelial cells upregulate adhesion molecule expression, promoting the recruitment of monocytes into the subendothelial space. Recruited monocytes ingest modified lipid and become foam cells, hallmarks of early atherosclerosis, trapped in the vessel wall. Progressive lipid accumulation and leucocyte recruitment leads to the gradual formation of an atheroma that protrudes into the lumen of the vessel wall, narrowing the artery. In addition to monocytes, other leucocyte populations including T lymphocytes, dendritic cells and mast cells have been implicated in the pathogenesis of atherosclerosis. As lesions progress, smooth muscle cells proliferate and migrate into the intima where they deposit extracellular matrix components and form a fibrous cap over the lesion. Rupture of unstable lesions causes thrombus formation, which may lead to myocardial infarction. These processes are now recognised to involve components of both the innate and adaptive immune systems [5].

Innate immunity constitutes the first line of defence against invading pathogens, and it is programmed to detect highly conserved molecular motifs called pathogen-associated microbial patterns (PAMPs) via specialised receptors. Amongst several families of pattern-recognition receptors (PPRs), Toll-like receptors (TLRs) are the most characterised so far. Although the exact gene numbers may differ between species, at least 13 different TLRs have been identified in mammals, each one with a certain degree of specificity for a range of ligands (reviewed later). 

The members of the TLR family share the same cytoplasmic domain with the interleukin (IL)-1 receptor, known as the Toll/IL-1R (TIR) homologous domain. As a result, TLRs activate signalling pathways shared with IL-1. The TIR domain recruits the adaptor protein myeloid differentiation primary response gene 88 (MyD88), which activates a family of IL-1R associated kinases (IRAKs). IRAKs in turn activate tumour necrosis factor receptor associated factor 6 (TRAF6), and elicit downstream signalling via the nuclear factor *κ*B (NF*κ*B) pathway. NF*κ*B translocation to the nucleus activates transcription of proinflammatory genes, including tumor necrosis factor (TNF)-*α*, IL-1, and IL-12. The MyD88-dependent pathway is shared by all TLRs, with the exception of TLR3. TLR4 signalling encompasses both the MyD88- and the MyD88-independent pathway. The MyD88-independent pathway, engaged by TLR-3 and -4, relies on TIR-domain-containing adaptor protein inducing interferon *β* (TRIF) to mediate interferon regulatory factor (IRF)-3 and NF*κ*B activation. TLR4 utlises Trif-related adaptor molecule (TRAM) to interact with TRIF and engage the MyD88-independent pathway [6].

In this paper, we will describe the expression, ligands, and functions of toll-like receptors in particular in reference to atherosclerosis. The aspects of TLR signalling will be treated in detail in other reviews in this series. We will consider toll-like receptors in both the human and murine systems highlighting important differences between the two organisms with regard to inflammatory mechanisms and TLR biology, which may hinder extrapolation of murine data into human systems.

## 2. Expression of Toll-Like Receptors

Cells of the innate immune system including monocytes/macrophages and dendritic cells are the main cellular expressers of toll-like receptors. However cells of the adaptive immune system and nonimmune cells have also been shown to express TLRs. Studies attempting to detail TLR expression on different cell types have some common shortcomings. Firstly, they tend to rely on expression at the mRNA level, due to limitations of existing antibodies. Secondly, discrepancies between TLR gene expression and responsiveness to TLR ligands are often observed. Thirdly, gene expression may be influenced by contamination with other cell types when, for example, purified populations of leucocytes are studied. 

**Figure 1:**
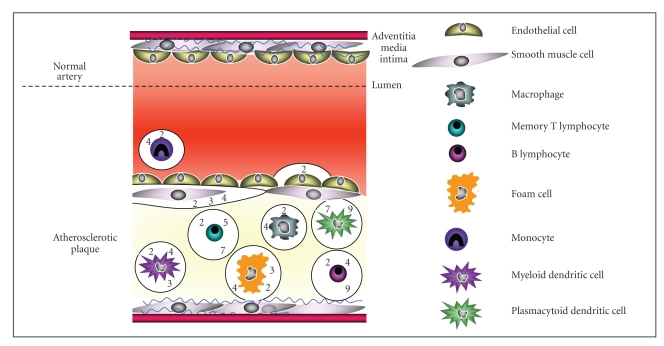
Toll-like receptor expression in atherosclerotic lesions is cell-type specific. Endothelial cell activation leads to increased expression of adhesion molecules, promoting the infiltration of monocytes into the subendothelial space. Recruited monocytes differentiate into macrophages, ingest lipid and become foam cells that are retained within the lesion, promoting plaque growth. Smooth muscle cells proliferate and migrate into the intima where they form a fibrous plaque over the necrotic core of the lesion. In addition, myleloid dendritic cells, plasmacytoid dendritic cells and lymphocytes are observed in lesions. Both immune and resident vascular cells in atherosclerotic arteries express a variety of toll-like receptors or increase their expression during disease development. Each cell type expresses a specific combination which might dictate its ability to respond to exogenous or endogenous ligands and the consequences of such stimulation. Ligation of toll-like receptors on cells within atherosclerotic plaques can lead to numerous downstream effects including; promoting monocyte recruitment, activation of plaque cells, induction of foam cell formation and activation of adaptive immune responses, which can all affect lesion development.

Notwithstanding these limitations, it is important to fully discern the expression patterns of TLRs in both health and disease as their knowledge may influence the choice of receptor or signalling pathway for therapeutic interventions targeting TLRs. This is particularly pertinent in atherosclerosis, a complex disease involving multiple inflammatory and noninflammatory cells. Within atherosclerotic lesions monocytes/macrophages, B and T lymphocytes, dendritic cells, smooth muscle cells and endothelial cells have all been described as expressing TLRs (Figure 1), or increasing their expression during disease development. Increased expression of TLR-1,-2, and -4 is found in inflammatory cells (including CD68-positive macrophages), smooth muscle cells and adventitial fibroblasts in human atherosclerotic vessels [7–10]. Consistent with human atherosclerosis, both TLR2 and TLR4 expression is increased in low-density lipoprotein receptor deficient (LDLR−/−) and apolipoprotein E deficient (ApoE−/−) mice, murine models of atherosclerosis [10, 11]. This increase in TLR expression by cells during atherogenesis may result in enhanced signalling through the TLRs and thus an exacerbation of cell activation and proatherogenic downstream pathways.

### 2.1. Monocytes/Macrophages

Monocytes and macrophages are present at all stages during atherogenesis and, due to their heterogenity, have numerous functions through which they affect atherosclerotic plaque initiation and development. Monocytes comprise 5–10% of peripheral blood leukocytes in both humans and mice. Interestingly, this is the only similarity between human and mouse blood. In humans, neutrophils comprise 50–70% and lymphocytes the remaining 30–50% of blood leucocytes. In contrast, lymphocytes are the main leucocyte component of murine peripheral blood comprising 75–95%, with neutrophils only accounting for 10–25% of peripheral blood leucocytes (reviewed in [12]). It has been shown recently that blood is not the only compartment where monocytes reside. Swirski et al. observed that the spleen can act as a reservoir of undifferentiated monocytes, which, upon ischemic myocardial injury, can relocate in injured tissue, and participate in wound healing [13].

Two major subsets of monocytes have been described in both humans and mice [14–16]. These subsets can be delineated on the basis of size, granularity and the differential expression of chemokine receptors and adhesion molecules. In humans, “classical” monocytes, which represent 90–95% of the total population of circulating blood monocytes, can be identified by high expression of CD14 and by a lack of CD16 (FC*γ*RIII) expression. These monocytes also express CCR2, CXCR2, CD62L, and CD64 [15]. In contrast, the other major subset of human monocytes, which have been shown to be similar to tissue macrophages, are CD14^low^CD16+ and express high levels of HLA-DR (MHCII) and CX3CR1 but do not express CCR2 or CD62L [17–19]. An intermediate subset of human monocytes that expresses high levels of CD14 and that are CD16 positive has also been described [20]. 

The two major subsets of murine monocytes resemble those that have been described in humans. The “inflammatory” subset of murine monocytes can be defined by their high expression of Ly6C/Gr1 and CCR2. In addition, these monocytes express CD62L and low levels of CX3CR1, which makes them similar in phenotype to the “classical” CD14+CD16− human monocyte subset [14]. The other major murine monocyte subset expresses low levels of Ly6C/Gr1 and high levels of CX3CR1. These monocytes do not express CCR2 or CD62L and thus resemble CD14^low^CD16+ human monocytes [14]. In contrast to humans, the two main monocyte subsets appear to be equally represented in murine blood. High-cholesterol feeding leads to an altered balance of the two major circulating monocyte subsets in ApoE−/− mice. Both Swirski et al. and Tacke et al. have demonstrated that high-cholesterol feeding of ApoE−/− mice leads to monocytosis of the Ly6C^high^ monocyte subset [21, 22]. These “inflammatory” monocytes are preferentially recruited into murine atherosclerotic plaques [22]. Monocytes are continuously recruited to atherosclerotic lesions, and their recruitment is proportional to lesion size [23].

Human blood monocytes express TLR1, TLR2, TLR4, TLR5, TLR6, TLR8, and TLR9 mRNA with TLR2 and TLR4 being the most highly expressed [24–27]. Surface expression of TLR2 and TLR4 has been confirmed by flow cytometry [26] and both the TLR2 ligand peptidoglycan and the TLR4 ligand LPS induce monocytes to secrete proinflammatory cytokines [25]. Circulating monocytes from patients with arterial disease exhibit increased expression of TLR4 and TLR2 compared to healthy controls [28–31]. However, such increases in expression do not always result in enhanced TLR signalling [32–34]. Analagous with human coronary artery disease, ApoE−/− mice with advanced atherosclerotic disease also display increased surface expression of TLR2 and TLR4 on circulating monocytes [35]. TLR4 expression in atherosclerotic lesions in ApoE−/− mice has been shown to colocalise with macrophage staining [10]. Increased expression of TLR2 and TLR4 in lesions may be a consequence of exposure to oxidised low-density lipoproteins in the plaque as expression of both receptors has been shown to increase *in vitro* following oxidised LDL stimulation and foam cell formation in monocyte-derived macrophages and THP1 cells [10, 36]. As we will discuss later, oxidised LDL can also act as a ligand engaging TLR-4, inducing a vicious circle of cell activation.

Despite the description of two subsets of human CD14+ peripheral blood monocytes with different LPS responsiveness [37], to our knowledge, no study has examined the differential expression of TLRs on different monocyte subsets. The varied and key functions of monocytes/macrophages in all phases of atherogenesis highlights the need for better understanding of the innate immune receptors expressed by these cells and their activation, in particular in relation to the different subsets of monocytes that have been described.

### 2.2. Dendritic Cells

Two broad subsets of dendritic cells have been described: myeloid dendritic cells (mDCs) and plasmacytoid dendritic cells (pDCs), although DC heterogeneity is greater than this basic simplification (reviewed in [38]). These two subsets, however, are relevant as they significantly differ in terms of TLR expression.

In the intima of normal arteries, networks of dendritic cells have been described [39–41]. According to Wick and colleagues, these dendritic cells form part of a vascular-associated lymphoid tissue (VALT), which also comprises T lymphocytes, macrophages and mast cells [42]. The function of VALT is postulated to be to monitor potential danger signals in arteries [42]. Dendritic cells in VALT are similar to skin Langerhan cells and are CD1a^+^S-100+lag+CD31−CD83−CD86− [41]. These intimal DC networks have also been observed in wild-type mice [43, 44]. In particular, dendritic cells have been identified in the arterial intima at atherosusceptible sites such as branch-points [41, 43, 45], suggesting that DCs may play a role in the initiation of atherosclerosis. Whether this role is beneficial or deleterious is not yet known.

mDCs express numerous TLRs at the mRNA level including TLR2, TLR3, TLR4, TLR5, TLR6, TLR7, and TLR8 [46–48]. Furthermore, mDCs secrete cytokines and upregulate costimulatory molecule expression in response to stimulation with the TLR ligands Poly(I:C), LPS and R848 [46–48]. Monocyte-derived dendritic cells (MoDCs) may be obtained by in vitro culture of monocytes in the presence of IL-4 and GM-CSF [49]. Similar to mDCs, monocyte-derived dendritic cells express mRNA for TLR2, TLR3, TLR4, and TLR5 and additionally express TLR1 mRNA [24]. Monocyte-derived DCs exhibit a strong response to LPS stimulation [50] and also respond to TLR3 stimulation with Poly(I:C) [48] by producing cytokines.

In contrast to mDCs, pDCs strongly express both TLR7 and TLR9 mRNA and only weakly express TLR2 and TLR4 mRNA [47, 50, 51], which may allow these cells to be particularly responsive to viruses. pDCs are activated, mature and secrete cytokines following stimulation with the TLR9 ligand CpG [25, 27, 47, 50, 51]. Similar to mDCs, pDCs also functionally respond to stimulation with the TLR7 ligand R848. However the engagement of TLR-7 in mDCs and pDCS leads to different functional outcomes: pDCs express IFN*α*, while mDCs express IL-12 in response to R848 stimulation [46].

In contrast to human mDC and pDC subsets, TLR1, TLR2, TLR4, TLR6, TLR8, and TLR9 have been shown to be expressed at the mRNA level by both murine DC subsets [52]. Further dividing the murine splenic mDCs into CD8+ and CD8− populations reveals that CD8+ mDC lack TLR5 or TLR7 expression but express more TLR3 in comparison to CD8− mDCs. Functionally, murine pDC and CD8− mDCs respond to ligands for TLR7 and TLR9 and CD8+ mDC respond to a TLR9 ligand only, by producing cytokines and increasing surface expression of co-stimulatory molecules [52]. Interestingly, dyslipidemia has been shown to functionally inhibit the CD8*α* ^−^ subset, and their ability to respond to TLR ligands [53]. As the CD8 subsets have not been identified in humans [12], it is unclear whether such differences are relevant in terms of human disease. It is also unclear whether differences between human and mouse DCs reflect true species-specificity or the fact that in most studies human dendritic cells are normally obtained from peripheral blood whereas murine dendritic cells are generally isolated from spleen. Further studies are needed to clarify these points.

Both pDCs and mDCs have been observed in human carotid atherosclerotic plaques, particularly in shoulder-regions, which are areas of plaque growth and instability, and at the base of the plaque [54, 55]. CD11c+ dendritic cells are recruited to atherosclerotic lesions via the chemokine fractalkine in murine models [44]. The precise role of dendritic cells in atherosclerosis is not yet clear. Different expression patterns of TLRs have been described for pDCs and mDCs and thus both subsets may contribute to atherogenesis differentially through recognition and response to different TLR ligands. pDCs in human plaques are high-producers of IFN*α* following TLR-9 stimulation [55]. Patients with acute coronary syndromes have lower levels of circulating mDCs, probably due to increased recruitment at the lesion site and secondary and tertiary lymphoid organs [56].

### 2.3. T Lymphocytes

T lymphocytes (both CD4+ and CD8+) are present in human and murine atherosclerotic lesions [57–60]. T cell clones isolated from human atherosclerotic plaques have been shown to be immunospecific for self-antigens including oxidised LDL [61]. Furthermore, the transfer of CD4+ T lymphocytes from ApoE−/− mice into ApoE−/− SCID mice has been shown to aggravate atherosclerotic lesion development and T lymphocyte accumulation in lesions [62]. In contrast, regulatory T cells have an athero-protective role in lesion development [63, 64].

At the mRNA level, TLR1, TLR2, TLR3, TLR4, TLR5, TLR7, and TLR9 have been detected in human peripheral blood T lymphocytes [27, 65] and flow cytometry has confirmed protein expression of TLR1, TLR2, TLR4, and TLR9 [66]. Differences in TLR expression patterns between T lymphocyte subsets and locations has been described, which may reflect specialised immune functions. Surface expression of TLR2 has been shown to increase following T cell receptor (TCR) activation [67] and memory T cells display enhanced responses to TLR-2, TLR-5 and TLR-7 activation compared to naïve T cells [65]. Tonsillar CD4+ T cells express more TLR1 and TLR9 than CD8+ T cells whereas CD8+ cells express more TLR3 and TLR4 than CD4+ cells [68]. In conjunction with TCR activation, ligands for TLR2, TLR3, TLR5, TLR7/8 and TLR9 act as costimulators for promoting proliferation and cytokine production by human T lymphocytes [65, 67, 69–71]. Interestingly, although both *α* *β* and *γ* *δ* T lymphocytes express TLR3, only stimulation of *γ* *δ* T lymphocytes with PolyIC, in association with TCR activation, leads to increased IFN*γ* secretion [71].

Murine T lymphocytes also express TLRs and respond to their ligands although discrepancies in the literature exist. Gelman et al. reported that activated splenic CD4+ T cells express and respond to ligands for TLR3 and TLR9 but not TLR2 and TLR4 [72]. However, Sobek and colleagues showed splenic murine T cells to express and respond to TLR2 following activation [73]. Different mouse strains were used in these studies, which may account for the differences observed. In addition to mRNA expression of TLR1, TLR2, TLR6, TLR7, and TLR9, murine CD8+ cells have also been shown to be responsive to TLR2 ligands with receptor ligation lowering the threshold for activation by antigen-presenting cells [74]. 

Murine CD4+CD25+ T regs express mRNA for TLR1, TLR2, TLR4, TLR5, TLR6, TLR7, and TLR8. Exposure of murine CD4+CD25+ regulatory cells to LPS leads to increased expression of activation markers, enhanced proliferation and augmented suppressor activity [75]. Costimulation of human CD4+CD25+ regulatory T cells with the TLR5 ligand flagellin increases the suppressive capacity of these cells [70]. The suppressor function of murine CD4+CD25+ cells is also increased following TLR7 stimulation [76].

### 2.4. B Lymphocytes

B lymphocytes express numerous TLRs at the mRNA and protein level. Human B cells express TLR1, TLR6, TLR7, TLR9, and TLR10 [27, 68, 77, 78] and secrete cytokines such as IL6 and TNF*α* in response to stimulation with CpG oligonucleotides [27, 78, 79] although discrepancies in the literature exist. Different patterns and levels of TLR expression have been described depending on the location and maturity of B lymphocytes [79]. For example, TLR2 is functionally expressed by a small subset of circulating B cells with intermediate CD19 expression and most tonsillar B cells [68, 80]. Naïve B cells express low levels of most TLRs but expression is increased upon activation and on memory B cells [79].

Naïve murine B cells express a wide repertoire of TLRs and proliferate in vitro in response to ligands for TLR2, TLR7, TLR9 and TLR4 [81–83]. In contrast to human B lymphocytes, murine B cells express TLR4 and respond to stimulation with LPS [82]. Furthermore, TLR expression levels on naïve murine B cells and memory B cells does not appear to differ as has been reported for their human counterparts [82].

### 2.5. Mast Cells

Mast cells are long lived tissue resident cells that derive from progenitors in the bone marrow, and circulate as progenitors in the peripheral blood until they are recruited to specific tissues where they undergo maturation [84]. Mast cells have important roles in host defence to helminth, bacterial and viral infections, and in allergic reactions. Upon activation, they release a variety of preformed mediators such as histamine, cytokines and proteases. Increased numbers of mast cells are observed at sites of plaque erosion, rupture, and haemorrhage in human atherosclerotic plaques, suggesting a role in the pathogenesis of thin cap fibroatheroma (TCFA) or vulnerable plaques [85]. Crossing mast cell deficient mice (Kit^W-sh/W-sh^) with LDLR−/− mice identified the requirement for mast cells in plaque development and inflammatory cell infiltration via mast cell IL-6 and IFN-*γ* induced protease production by endothelial and smooth muscle cells [86]. Human and rodent mast cells have been shown to express TLR-1 to -7 and TLR-9 [87].

### 2.6. Resident Vascular Cells

Expression of several TLRs can be found on normal human vessels. However primary arterial endothelial and smooth muscle cells have been shown to respond to a wider range of TLR ligands than these cell types from venous tissues [88]. In addition, differential expression of TLRs in vessels occurs across different vascular beds. For example, TLR3 mRNA is expressed in the aorta whereas the temporal and iliac arteries do not express TLR3 but instead express TLR8 mRNA. The carotid artery, however, expresses mRNA for both TLR3 and TLR8 [89]. In contrast to normal human vessels, which express relatively low-levels of TLRs, protein expression of TLR1, TLR2, and TLR4 is increased in human atherosclerotic vessels [7]. TLR-2 is expressed on endothelial cells in atheroprone regions [11], as we will discuss later in more detail. 

Human vascular smooth muscle cells constitutively express TLR1, TLR3, TLR4, and TLR6 at the mRNA level [90]. In addition, murine aortic SMCs constitutively express TLR2 mRNA [91]. However, TLR2 expression is inducible on human SMCs following exposure to *Chlamydia pneumoniae*, TLR3 and TLR4 ligands [92]. Expression of TLR4 on human vascular SMCs at the protein level has been shown [93, 94] and more importantly, functional expression of TLRs on smooth muscle cells has also been described. Exposure of aortic SMCs to the TLR4 agonist LPS induces MCP-1, IL6 and IL-8 production [91, 94, 95]. Stimulation of SMCs with the synthetic dsRNA ligand Poly:IC results in MCP1 and IL6 release [90] and exposure of SMCs to *Chlamydia pneumoniae* leads to TLR2-dependent MCP-1 release [92].

## 3. Toll-Like Receptor Ligands

A wide-repertoire of both exogenous and endogenous TLR ligands have been described (Table 1). TLRs 1, 2, 4, 5, and 6 specialise in the recognition of mainly bacterial products. TLRs 3, 7, 8, and 9, in contrast, specialise in the detection of viral and bacterial nucleic acids. For instance, TLR-2 is essential for the recognition of bacterial lipoproteins, and lipotheicoic acid. TLR-3 is implicated in recognition of viral double stranded (ds) RNA. TLR-4 is predominantly activated by lipopolysaccharide (LPS), while TLR-5 detects bacterial flagellin, and TLR-9 is required for responses to unmethylated CpG DNA typically of bacterial origin [6]. Viruses can also be recognized by TLR2 and TLR4. Very recently, it has been reported that TLR2 activation by viruses led to the production of type I interferon only in response to viral ligands in Ly6C^hi^ inflammatory monocytes [96].

Many exogenous TLR ligands are expressed in atherosclerotic lesions. Infectious agents, such as *Chlamydia Pneumoniae,* have been detected in atherosclerosis [97, 98]. Human atherosclerotic plaques contain numerous bacterial signatures, including nucleic acids [99], peptidoglycan [100], and exogenous heat shock proteins (HSP) [101]. Viruses have also been detected (reviewed in [102]). It is worth noting, however, that peptydoglican—derived molecules are also sensed by nucleotide oligomerization binding domain (Nod)-like receptor family members [103]. 

There is growing evidence that TLR signalling may be elicited in the absence of infection though “endogenous” ligands generated at sites of tissue remodelling and inflammation, as reviewed in [104]. The atherosclerotic plaque is characterised by accumulation of lipoproteins, extracellular matrix turnover during tissue remodelling, and finally formation of debris from necrotic cells in the necrotic core. As such, the atherosclerotic plaque is likely to contain many endogenous ligands (Table 1). For example, HSPs induce the production of proinflammatory cytokines in a TLR2- and TLR4-dependent pathway [105, 106]. Degradation products of extracellular matrix macromolecules are generated during tissue injury, or remodelling, and have been found to function as TLR ligands. The alternative splice of fibronectin, extra domain A (EDA) that has been shown to signal through TLR4 is detected in atherosclerotic plaques [107]. Tenascin C has recently been identified as a TLR-4 ligand with relevance in chronicity of inflammatory arthritis and given the similarities between RA and atherosclerosis, may also be relevant in atherosclerosis [108]. Hyaluronan (HA), one of the major glycosaminoglycans of the extracellular matrix that undergoes rapid degradation at sites of inflammation, is another ligand for TLR2 and TLR4 [109]. A recent study has documented that versican, a large extracellular matrix proteoglycan, can activate tumour-infiltrating myeloid cells through TLR-2 and its coreceptors TLR-6 and CD14 and elicit the production of proinflammatory cytokines including TNF-alpha that enhance tumor metastasis [110]. A similar mechanism may occur in infiltrating atherosclerotic plaque monocytes/macrophages.

Lipids are also putative ligands for TLR-2 and 4. Saturated fatty acids display the capacity of delivering a TLR4 signal and to induce inflammatory gene expression, while polyunsaturated fatty acids block the activation of TLR4 [111]. However, the ability of saturated fatty acids to directly induce TLR signalling has recently been questioned [112]. Minimally modified (mm) low-density lipoproteins (LDL) have been shown to induce cytokine production via TLR-4/MyD88 signalling [113] and reactive oxygen species via TLR4/MyD88-independent signalling [114] in murine macrophages. Very recently, oxidised LDL and amyloid-*β* peptide have been shown to initiate inflammatory responses through a TLR-4 and -6 heterodimer in association with CD36 [115]. Amongst phospholipids relevant to innate immunity, particular attention has been given to phosphorylcholine—a universal prokaryotic and eukaryotic membrane molecule, also represented in the phospholipid quota within lipoproteins. Watson et al. identified oxidised products of 1-palmitoyl-2-arachidonoyl-sn-glycero-3-phosphorylcholine (oxPAPC) as the major bioactive lipid in mmLDL [116]. Other oxidized phospholipid moieties contained within the fatty acid side chains, have been shown to elicit signalling through CD36 [117]—a class B scavenger receptor that mediates platelet aggregation and adhesion after injury, dendritic-cell recognition and uptake of apoptotic cells. Interestingly, CD36 acts as a coreceptor of TLR-2/6 heterodimers during recognition of microbial diacylglycerides [118]. In addition, the scavenger receptor lectin-like oxidised low-density lipoprotein receptor-1 (LOX-1) cooperates with TLR2 during cellular responses to *klebsiella pneumoniae* [119]. ApoCIII, a component of very-low-density lipoprotein (VLDL), was also found to be recognised by TLR2 and to induce proinflammatory signals in monocytes [120].

## 4. Functional Consequences of Toll-Like Receptor Activation in Atherosclerosis

### 4.1. TLRs Regulate Leukocyte Subset Recruitment and Activation in Atherosclerosis

Interestingly the first cells that display TLR expression in early atherosclerosis appear to be resident vascular cells such as the endothelium. Atherosclerotic lesions do not develop uniformly throughout the arterial system. Instead, lesions preferentially occur at sites of disturbed blood flow such as curvatures, branches, and bifurcations such as the lesser curve of the aortic arch [121]. TLR-2 expression is increased in endothelial cells placed at regions of susceptibility of atherosclerosis, such as the inner curvature, and it is associated with areas of monocyte recruitment in atherosclerosis-prone LDLR−/− mice [11]. However, whether endothelial cell expression of TLR2 precedes temporally the migration or is a consequence of monocyte recruitment and production of proinflammatory mediators, is unknown. 

The recruitment of cells belonging to innate and adaptive immunity from the circulation into the subendothelial space is a critical step in atherosclerotic lesion development. Over the last decade this process has been extensively studied and the sequence of events that lead to leucocyte recruitment have been termed “the adhesion cascade” [122]. Leucocytes tether and roll along the endothelium through low-affinity interactions that are mediated by the selectin family of adhesion molecules. Integrin activation via interactions between chemokines on the apical surface of endothelial cells and chemokine receptors on the leucocyte results in the firm adhesion of leucocytes to the endothelial cells and their arrest from flow. Firmly adherent leucocytes then migrate across the endothelial cell (EC) layer towards a chemotactic gradient by either passing through the borders between adjoining ECs (paracellular pathway) or by passing directly through the cytoplasm of ECs (transcellular pathway). The specific expression patterns of adhesion molecules and chemokines on both endothelial cells and leucocytes coupled with the dynamic regulation of these molecules allows highly regulated recruitment of different leucocyte subsets, that results in specific tissue responses. Activation of toll-like receptors induces the expression of adhesion molecules including selectins, chemokine and chemokine receptor genes and thus TLR signalling can regulate cell migration to sites of inflammation [88, 123–125].

Crossing of MyD88−/− mice with ApoE−/− mice has been shown to reduce the development of atherosclerotic lesions by approximately 60% and macrophage infiltration by 75% [126]. Whole body deficiency of TLR4 or TLR2 in ApoE−/− mice resulted in a 55% reduction of atherosclerotic lesion development [126, 127] and a 65% reduction in macrophage infiltration in ApoE−/−TLR4−/− mice [126]. In these studies, decreased lesion size is associated with a reduction in serum CCL2/MCP-1 levels [126–128]. Mullick et al. have shown that bone marrow transfer from TLR2−/− to LDLR−/− mice was effective in preventing exogenous TLR2 ligand-induced disease amplification, but not baseline atherosclerotic lesion formation [127]. Interestingly, C3H/HeJ mice, which carry a missense mutation affecting the cytoplasmic portion of TLR4, are resistant to diet-induced atherosclerosis [129, 130]. However, bone marrow transplantation from C3H/HeJ to Apolipoprotein E (ApoE)−/−, did not alter atherosclerosis development [131]. This finding points towards a key role for TLR expression in vascular cells [11]. Of relevance, only endothelial cells, but not myeloid cells, express TLR2 in murine lesions [11]. However, in human lesions TLR2 expression was detected in macrophages, endothelial cells and smooth muscle cells [7]. Differences in expression of TLR2 could result either from differences between early versus late disease stage, or a difference between murine and human atherosclerosis. 

Recruited macrophages can be activated by a large number of signals within an atherosclerotic lesion, including innate activation through TLRs. The nature of macrophage activation plays a key role in determining the phenotype and development of an atherosclerotic plaque. Plaque macrophages display features of activation and can exert numerous effects on other vascular cells via release of a host of proinflammatory mediators including tumour necrosis factor (TNF)-*α*, which leads to the engagement of the proinflammatory cytokine cascade, resulting in interleukin (IL)-1, and IL-6 production. In addition, activated macrophages play key roles in lipid uptake and plaque stability. All of these functions may be initiated or enhanced by toll-like receptor engagement. Indeed, we have recently shown in human atherosclerosis that TLR-2 and MyD88 play a predominant role in NF*κ*B activation, and in the production of inflammatory mediators, and matrix degrading enzymes in human atherosclerosis [132], suggesting that TLR-2 signaling influences the plaque vulnerability to rupture. In contrast, signalling though TLR-4 and the downstream TLR-4 signaling adaptor TRAM was not rate-limiting for cytokine production in human atherosclerotic plaques, but may have a role in MMP production.

### 4.2. TLR Engagement Influences Foam Cell Formation

Toll-like receptor pathways can influence lipid uptake by macrophages and thus foam cell formation. Stimulation of murine macrophages with TLR2, TLR4 and TLR9 ligands promotes lipid uptake and foam cell formation [110, 133–135]. *Chlamydia pneumoniae* stimulation of macrophages can induce foam cell formation via MyD88-dependent and MyD88-independent pathways downstream of TLR2 and TLR4 [136–138]. Expression of the scavenger receptors SRA, macrophage receptor with collagenous structure (MARCO) and lectin-like oxidised low-density lipoprotein receptor-1 (LOX-1) are upregulated by macrophages following TLR3, TLR4 or TLR9 stimulation [134, 139], which is one potential mechanism of enhanced foam cell formation following TLR stimulation. Almeida et al. recently showed a role for TLR2 in increased lipid body formation in mycobacterium bovis bacillus Calmette-Guerin infection [140]. In addition, TLR4-dependent fluid phase uptake (macropinocytosis) of lipids has been shown to occur in differentiated macrophages [141]. 

Fatty acid binding proteins including aP2 (FABP4) and Mal1 (FABP5) facilitate the uptake of fatty acids by cells. Activation of TLR2, TLR3, and TLR4 on murine macrophages leads to increased expression of aP2 [142] and TLR2 and TLR4 agonists increase murine macrophage Mal1 expression [143]. However, increased expression of aP2 and Mal1 are not observed in human macrophages following TLR stimulation [143], suggesting different mechanisms of regulation of these molecules. Agonists of TLR2, TLR3, TLR4 and TLR7 have also been shown to increase ADRP/ADFP expression, which is associated with the formation of lipid droplets [143, 144]. Overexpression of ADRP/ADFP has been shown to increase macrophage cholesterol ester storage [145]. 

TLRs and their ligands may also interfere with cholesterol efflux mechanisms. Cholesterol efflux may be achieved through genes including ATP-binding cassette transporter A1 (ABCA1) and G1 (ABCG1), which are regulated by lipid-X receptors (LXRs). Signalling pathways involving IRF3 downstream of TLR3 and TLR4 activation can lead to inhibition of LXR transcriptional activity and thus reduced expression of LXR target genes and consequently reduced cholesterol efflux [146]. Interestingly, LXRs can inhibit inflammatory signalling pathways following TLR stimulation in a MyD88-dependent mechanism [147]. Thus, TLRs may affect lipid uptake and accumulation in macrophages through several mechanisms.

### 4.3. TLRs May Control Antigen Presentation and T Cell Activation in Atherosclerotic Plaques

Proposed antigens in atherosclerotic plaques include oxidised LDL, oxidised phosphatidylcholine, heat shock proteins, beta-2-glycoprotein-1 and antigens of infectious organisms such as herpes virus, cytomegalovirus, and *Chlamydia pneumoniae*. The generation of an adaptive immune response starts with the encounter between an antigen presenting cell (APC) and an antigen in the peripheral tissues. This process requires the acquisition by DCs of a mature phenotype through upregulation of costimulatory molecules such as CD80, CD86 and CD40. TLR ligation typically induces expression of these costimulatory molecules (reviewed in [148]) in all DC subsets regardless of their differential TLR expression profiles.

DC maturation is followed by their migration to the draining lymph nodes. This migration is also mediated by TLR-induced downregulation of inflammatory chemokine receptors and upregulation of the receptors for lymphoid chemokines. CCR6 downregulation and CCR7 upregulation, is observed in experimental models of atherosclerosis [149]. This change in chemokine receptor expression is crucial for dendritic cells to migrate from the peripheral tissues to the T lymphocyte areas of draining lymph nodes. Besides secondary lymphoid organs, antigen presentation can happen in other sites in atherosclerosis. A variety of antigen presenting cells might perform antigen presentation within an atherosclerotic plaque, including professional dendritic cells and lesional macrophages. Recently, tertiary lymphoid organs are proposed to be alternative sites of antigen presentation within atherosclerotic vessels [150, 151].

The next step is the differentiation of naive CD4+ T lymphocytes into either T_H_1 or T_H_2 or T_H_17 cells [152]. The direction of differentiation is influenced by both the concentration of presented peptide and the presence of specific cytokines. For instance IL-12 and IL-18 tend to promote the generation of a T_H_1 response. T_H_1 responses appear to dominate in both humans and mice during atherogenesis and has been shown to be proatherogenic [153]. Exogenous treatment with either IL-12 or IL-18 accelerates atherosclerotic lesion development [154, 155] whereas deficiency of either IL-12 or IL-18 results in decreased lesion formation in ApoE−/− mice [156, 157]. Lesions in ApoE−/−IL-12−/− mice also display a more stable phenotype. Furthermore, both ApoE−/−IL-12−/− and ApoE−/−IL-18−/− mice exhibit a switch from T_H_1 to T_H_2 immunoglobulin subclass [157]. T_H_1 cells may exert proatherogenic actions in part through secretion of proinflammatory cytokines such as interferon gamma (IFN*γ*) and tumour necrosis factor (TNF)-*α* that can then activate macrophages, induce protease and inflammatory cytokine production and inhibit smooth muscle cell proliferation and collagen production [158]. Indeed, genetic deletion of IFN*γ* in LDLR−/− and ApoE−/− mice leads to decreased atherosclerotic lesion size [155, 159] as does deletion of its receptor IFN*γ*R in ApoE−/− mice, which also results in a more stable lesion phenotype [160]. Interestingly, genetic deficiency of MyD88, known to be associated with a decrease of atherosclerosis development [128], leads to impaired T_H_1 differentiation and a switch towards T_H_2 responses [161, 162]. 

Conversely, Th2 responses are broadly considered antiatherogenic. Extreme hypercholesterolemia itself in ApoE−/− mice has been shown to skew T cell responses towards a T_H_2 phenotype [163]. Examining atherosclerotic lesion development in ApoE−/− mice on either a C57BL/6 or BALB/c genetic background, which display opposing T_H_ responses revealed that ApoE−/− mice on a C57BL/6 genetic background, which have predominantly T_H_1 responses, develop significantly more atherosclerosis than ApoE−/− mice on a BALB/c genetic background, which have predominantly T_H_2 responses [164]. Furthermore, ApoE−/− mice on a BALB/c genetic background display decreased CD4+ T cell accumulation and reduced MHCII expression in atherosclerotic lesions compared to ApoE−/− mice on a C57BL/6 genetic background [164]. The T_H_2 cytokine interleukin (IL)-10, produced both by lymphocytes and macrophages, is antiatherogenic. IL-10-deficient (IL-10−/−) mice or LDLR−/− mice in which the leucocytes are IL-10−/− develop larger atherosclerotic lesions than matched controls [165, 166]. Lesions in IL-10−/− mice also exhibit increased accumulation of activated T cells, increased IFN*γ* secretion and decreased collagen production [165, 166]. Paradoxically, TLR-2 signalling, known to be proatherogenic, has been shown to promote T_H_2 differentiation [167, 168]. 

TLR-2 has also been linked to regulatory T cell responses. The synthetic bacterial lipoprotein Pam3Cys-SK4, a TLR-2 ligand, leads to expansion of regulatory T cells and a temporal inhibition of their suppressive activity [169, 170]. Recently, Manicassamy et al. have shown that TLR-2 stimulation of dendritic cells leads to an induction of T regulatory cells [171]. An athero-protective role for regulatory T cells in murine atherosclerosis, in part through suppression of T_H_1 responses has been described [64, 153, 172]. 

Very recent evidence is presenting a complex role of T_H_17 in atherosclerosis, which encompasses both modulatory [173] and proatherogenic functions of IL-17 [174]. Interestingly, SIGIRR (Single Ig IL-1-related receptor), a negative regulator of IL-1 receptor and Toll-like receptor signaling, has been shown to govern Th17 cell differentiation and expansion [175]. It also emerged recently that pDCs are capable of promoting Th17 differentiation in response to TLR7 stimulation [176].

Although few B lymphocytes have been observed in human atherosclerotic plaques [58], studies in mice have revealed a potential protective role for B lymphocytes in atherogenesis [177, 178]. B lymphocytes express both antigen-specific B-cell receptors and pattern-recognition receptors, including TLRs (described above). Ligation of TLRs on B lymphocytes has been shown to induce polyclonal activation and secretion of immunoglobulin M (IgM) antibodies [179, 180]. In addition, it has recently been shown that activation of TLR-2 and TLR-4 on murine B1 cells results in enhanced production of IgM antibodies against oxidation-specific epitopes [181]. Interestingly, serum IgM has been described as atheroprotective in LDLR−/− mice as LDLR−/− mice deficient in serum IgM exhibit larger lesions with increased cholesterol crystal formation [182].

### 4.4. TLRs as Therapeutic Targets in Atherosclerosis

Given the large body of data suggesting that TLRs can contribute to several atherosclerotic mechanisms key to the inititation and development of lesions such as leucocyte recruitment and foam cell formation (discussed above), these molecules may be important targets for the development of novel antiatherogenic therapeutics. To date, TLR-2 and TLR-4 have been the best characterised in terms of their contribution to atherosclerotic lesion development. Antagonism of TLR-2 or TLR-4 signalling is currently perceived as the most attractive target for development of therapeutics for the treatment of atherosclerosis. Indeed, deletion of either TLR-2 or TLR-4 confers a similar degree of protection from lesion development in murine models of atherosclerosis [126, 127]. However, as discussed, there are many differences between the human and murine immune systems, including the cellular expression patterns of TLRs. This may hinder the extrapolation of targets from murine studies into human targets. Indeed, we have recently demonstrated that a TLR-2 blockade can inhibit cytokine, chemokine and MMP production in human atherosclerosis, while interruption of TLR-4 signalling did not have a significant impact on the production of proatherogenic mediators [132]. Additionally, in a murine model of myocardial ischemia/reperfusion injury, TLR-2 blockade has recently been shown to reduce infact size and maintain heart function through reduction of proinflammatory mechanisms [183]. Together these studies support the idea that TLR-2 blockade may be beneficial in cardiovascular disorders. It is possible to envisage that blockade of TLR-2 during acute phases of the disease could be preferred to its chronic use. As TLRs are essential components of both the innate and adaptive immune responses and as they are expressed on both resident vascular and leucocyte populations, further studies are needed to ascertain the most effective timing of TLR inhibition in cardiovascular disease.

## 5. Conclusions

The role of TLRs in the development of atherosclerosis has just started to be unravelled. The key immune and resident vascular cells in the initiation and development of atherosclerosis all express various TLRs, suggesting these receptors and their ligands are critical players in atherogenesis. So far, it appears that TLR-2 and -4 activation has profound consequences on the recruitment of monocytes and foam cell formation in murine models of atherosclerosis. TLR-2 signalling appears to be a predominant event for activation of inflammation and matrix degradation in human atherosclerotic lesions. The consequences of activation and blockade of other TLRs in atherosclerosis remains to be explored. Due to the intricate outcomes of activation of TLRs on adaptive immunity, further studies need to explore the relationship between innate and adaptive responses in atherosclerosis.

## Figures and Tables

**Table 1:** Exogenous and Endogenous TLR ligands. Ligands in italics represent ligands for which a link to atherosclerosis has been identified.

TLR Receptor	Exogenous Ligand	Endogenous Ligand
TLR1	Mycoplasma tri-acyl lipopeptides [184], N.meningitides soluble factors (with TLR2) [185]	

TLR2	*Pam3CSK4* (synthetic TLR2/TLR1 agonist) [127],	Necrotic cells [186–189],
	Mycobacterial lipoprotein (with TLR1) [190],	*Apoliprotein CIII * [120],
	Bacterial lipoproteins (with TLR6) [191, 192]	*Oxidised LDL* [36],
	Yeast carbohydrates, [192]	Serum amyloid A [193],
	Borrelia burgdorferi lipoprotein (with TLR1) [194]	Amyloid beta [195]
	Staph epidermidis phenol-soluble modulin [196]	Versican [110],
	Viral envelope glycoproteins [197, 198]	
	*Peptidoglycan* (Gram + bacteria) [199]	
	Glycoinositolphospholipids (Trypanozoma cruzi),	
	Glycolipids (Treponema maltophilum),	
	Porins (Neisseria), Zymosan (fungi),	
	Atypical LPS (Leptospira interrogans and *Porphyromonas gingivalis*) [200–202]	

TLR2/TLR4	HSP60 [203],	*HSP60, HSP70, Gp96, HMGB1, * [204–211]
	*Chlamydia pneumoniae* [138],	*Hyaluronan fragment* [109, 212, 213]
	*HSP60 from Chlamydia pneumoniae* [106],	*Biglycan* [214]
	*Porphyromonas gingivalis* [215]	

TLR3	*Viral dsDNA* [48, 90, 216]	*mRNA* [217]

TLR3/TLR9	*CMV* [218, 219]	

TLR4	*Lipopolysaccharide,* [220–224]	Lung surfactant protein-A, [225]
	Viral envelope glycoproteins, [226, 227]	Tenascin C, [108]
	Taxol (plant), RSV fusion protein, MMTV envelope proteins, [200]	*Fibrinogen,* [228, 229]
	*HSP60 from Chlamydia pneumoniae* [93, 105]	*Fibronectin EDA,* [230]
		*Heparan sulphate,* [231–233]
		*Beta-defensin 2,* [234] [235]
		*Minimally-modified LDL,* [113, 236]
		*Oxidised LDL,* [10]
		Amyloid beta peptide and oxididised LDL [115]

TLR5	Bacterial flagellin [237, 238]	

TLR6	Mycoplasma di-acyl lipopeptides [239],	
	Group B Strep heat-labile soluble factor, Staph phenol-soluble modulin [200]	

TLR7	Various synthetic compounds including imidazoquinoline, loxoribine and bropirimine [200]	

TLR7/TLR8	Single stranded RNA [240–242]	

TLR7/TLR9		Nucleic acid-containing immune complexes [243–245]

TLR9	Hypomethylated CpG motifs in microbial DNA [241, 246, 247],	
	*HSV-2* [241]	
